# Erratum for Møllebjerg et al., “The Bacterial Life Cycle in Textiles Is Governed by Fiber Hydrophobicity”

**DOI:** 10.1128/spectrum.02880-22

**Published:** 2022-08-18

**Authors:** Andreas Møllebjerg, Lorena Gonzales Palmén, Klaus Gori, Rikke Louise Meyer

**Affiliations:** a Interdisciplinary Nanoscience Center, Aarhus University, Aarhus, Denmark; b Department of Bioscience, Aarhus University, Aarhus, Denmark; c WATEC Aarhus University Centre for Water Technology, Aarhus University, Aarhus, Denmark; d Novozymes A/S, Bagsvaerd, Denmark

## ERRATUM

Volume 9, no. 2, e01185-21, 2021, https://doi.org/10.1128/Spectrum.01185-21. Page 5, Fig. 5: Due to an error during upload of the final files for publication, Fig. 5 is an inadvertent duplicate of Fig. S4 in the supplemental material, which is very similar. The correct Fig. 5 is shown below.

**Figure 1:**
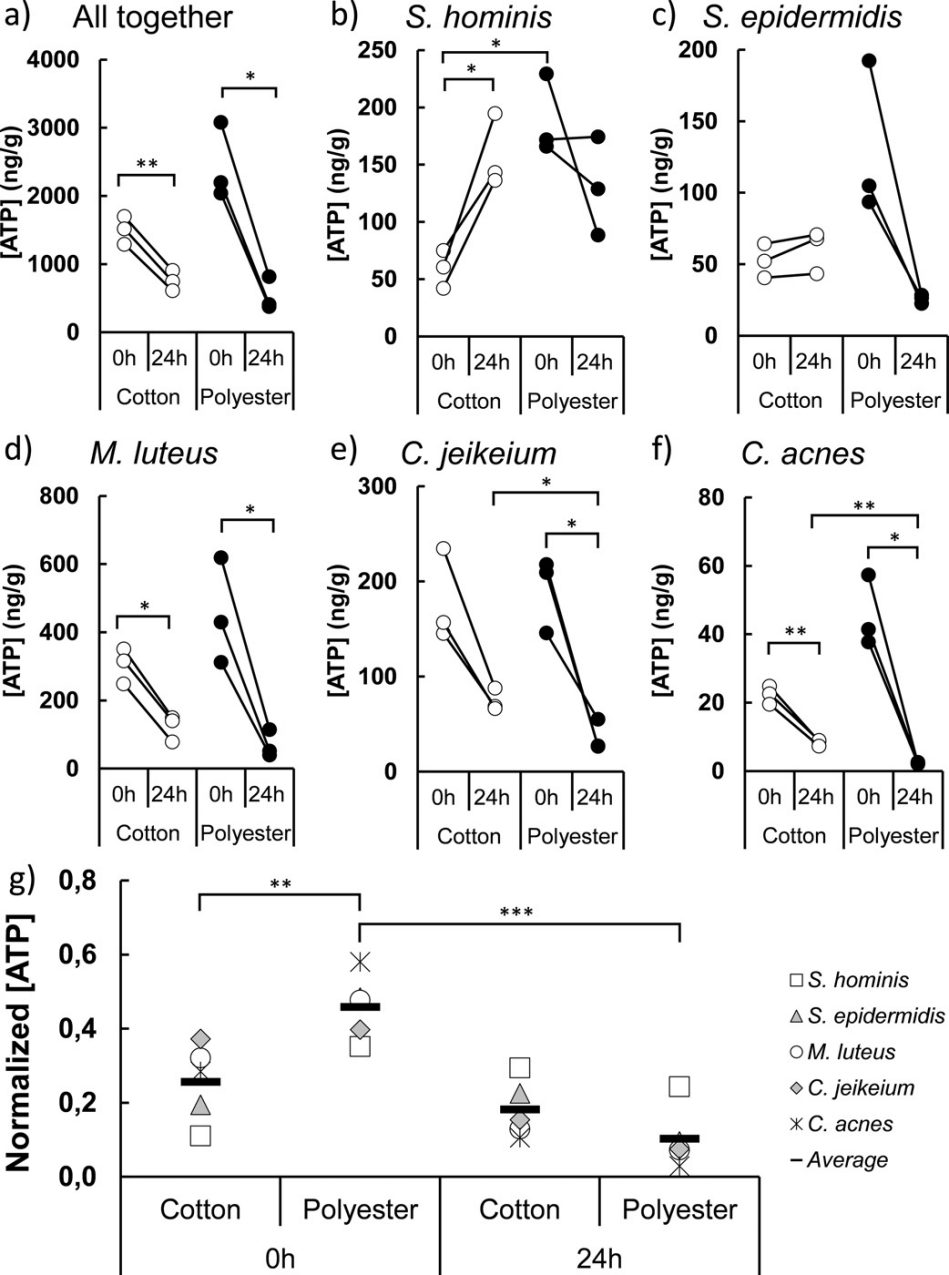


Correction of this figure does not change the conclusions of this paper.

